# OsEUL Lectin Gene Expression in Rice: Stress Regulation, Subcellular Localization and Tissue Specificity

**DOI:** 10.3389/fpls.2020.00185

**Published:** 2020-03-02

**Authors:** Jeroen Lambin, Sinem Demirel Asci, Malgorzata Dubiel, Mariya Tsaneva, Isabel Verbeke, Pieter Wytynck, Jeroen De Zaeytijd, Guy Smagghe, Kondeti Subramanyam, Els J. M. Van Damme

**Affiliations:** ^1^Department of Biotechnology, Faculty of Bioscience Engineering, Ghent University, Ghent, Belgium; ^2^Department of Plants and Crops, Faculty of Bioscience Engineering, Ghent University, Ghent, Belgium

**Keywords:** abscisic acid, *Euonymus*, lectin, nucleocytoplasmic localization, rice, stress regulation

## Abstract

The *Euonymus* lectin (EUL) family is a unique group of carbohydrate-binding proteins that is omnipresent in plants. Sequences encoding EUL-related lectins have been retrieved from all completely sequenced plant genomes. The rice (*Oryza sativa*) genome contains 5 functional EUL genes referred to as *OsEULS2*, *OsEULS3*, *OsEULD1a*, *OsEULD1b*, and *OsEULD2*. In this study we focused on the tissue specific expression, stress inducibility and subcellular localization of the rice EULs. Even though the EUL domain sequence is highly conserved among the rice EULs (at least 80% sequence similarity) different biotic and abiotic stress treatments yielded unique responses for the different EULs. Transcript levels for OsEULs were differentially affected by drought and salt stress, ABA treatment, pathogen infection or insect infestation. Analysis of promoter activity revealed differential expression and tissue specificity for the 5 OsEUL genes, with most expression observed in the vascular system of roots and shoots, as well as in the root tips and seeds. At cell level, all OsEULs are located in the nucleus whereas OsEULD1b and OsEULD2 also locate to the cytoplasm. This paper contributes to the functional characterization of the EULs and provides insight in the biological importance of this family of proteins for rice.

## Introduction

Lectins are defined as proteins containing one or more lectin domains that bind carbohydrate structures in a reversible, specific and non-enzymatic way. These carbohydrate structures can occur as monosaccharides, polysaccharides or as glycan structures attached to proteins or lipids (Van Holle and Van Damme, 2018). Although lectins are present in all kingdoms of life, most carbohydrate-binding proteins have been characterized from plants. The whole group of plant lectins is highly diverse in sequence, molecular structures, biochemical properties and biological activities. Nevertheless, lectins can be classified in different families based on the presence of particular lectin motifs. In the 1980s and 90s plant lectin research focused on vacuolar lectins that were abundant in storage tissues and could easily be purified using affinity chromatography. Breakthroughs in molecular biology, genomics, transcriptomics and bio-informatics resulted in the discovery of a new class of low abundant, nucleocytoplasmic lectins that are specifically upregulated after plant exposure to biotic and/or abiotic stimuli, implying the involvement of these lectins in stress signaling. For example, the tobacco lectin, referred to as Nictaba, is only detectable after treatment of tobacco leaves with methyl jasmonate (MeJA), cold stress and herbivory by caterpillars (Delporte et al., 2015). Overexpression of Nictaba-like lectin genes from *Glycine max* confers tolerance toward *Pseudomonas syringae* infection, aphid infestation and salt stress in transgenic *Arabidopsis* plants (Van Holle et al., 2016). Another well studied lectin is Orysata, a rice lectin that is up-regulated after several abiotic stresses such as salinity, drought stress and abscisic acid (ABA) treatment (De Souza Filho et al., 2003). Overexpression of the *Orysata* gene resulted in rice plants with higher salt tolerance (De Souza Filho et al., 2003; He et al., 2017).

Recent studies aiming to make an inventory of the occurrence of lectin motifs in a few model species with a fully sequenced genome revealed that most lectin sequences encode multi-domain proteins in which one or more lectin domains are linked to other protein domains such as a protein kinase domain, an F-box domain or a glycosyl hydrolase domain. However, some lectin motifs, such as the *Euonymus* lectin domain have not been found in association with other known protein domains (Van Holle et al., 2017). The family of *Euonymus*-related lectins (EUL) is unique in that it is ubiquitous in the plant kingdom. EUL sequences have been retrieved from all annotated genomes of land plants. EUL sequences are composed of one or two EUL lectin domains, further referred to as S (single) type EULs and D (double) type EULs (Fouquaert et al., 2009). The *Arabidopsis thaliana* genome contains only one member of the EUL family, named *ArathEULS3*, which interacts preferentially with galactosylated glycans. Transcript levels for *ArathEULS3* are enhanced when plants are subjected to drought, salt stress, ABA treatment or after infection with *P. syringae* (Van Hove et al., 2015).

In contrast to many dicot species the monocots contain a mixture of S- and D-type EUL sequences. The rice genome contains two S-type EULs (called *OsEULS2* and *OsEULS3*) and three D-type EULs (called *OsEULD1a*, *OsEULD1b* and *OsEULD2*). The EUL domain sequence is highly conserved among the rice EULs (at least 80% sequence similarity) (De Schutter et al., 2017). Despite the high sequence similarity between OsEULs the proteins have very different carbohydrate-binding activities. For instance, OsEULS2 has a preference for high-mannose N-glycans (Al Atalah et al., 2012) while OsEULD1a rather binds to galactose related sugars (Al Atalah et al., 2014). Analyses of the promoter region of the five rice EUL genes revealed a variety of stress responsive elements (De Schutter et al., 2017). RNA sequencing data and transcriptomics studies also indicate that transcript levels for EULs change after exposure of the plant to stress conditions. At present only a few scattered reports of proteomics data suggest that at least some of the OsEULs are stress-inducible proteins. For instance, a proteomics study for rice plants reported enhanced levels for some of the rice EULs after salt, ABA and drought treatment (Moons et al., 1995; Rabello et al., 2008).

In the past two decades several stress inducible plant lectins have been reported in literature. However, only few stress inducible lectins have been studied and functionally characterized. In this paper we focused on the tissue-specific expression, the subcellular localization and the stress inducibility for a family of closely related EULs from rice. Despite the high sequence similarity between the lectin domains important differences are observed for the five OsEULs. The experimental data confirm the stress dependent regulation of these lectins and provide insight in the biological importance and possible functions for rice EULs.

## Materials and Methods

### Plant Material

*Oryza sativa japonica* cv Nipponbare seeds were dehusked and sterilized for 5 min in 70% ethanol followed by incubation in 5% NaOCl containing 2–3 drops of Tween 20 under constant shaking at 150-180 rpm for 45 min. Afterwards the seeds were rinsed thoroughly with sterile water and incubated overnight with constant shaking at 150–180 rpm to synchronize the germination process.

### Abiotic Stress Treatment

For all abiotic stress experiments, seed germination was performed on a sterile filter paper soaked in half strength liquid (MS) medium (Murashige and Skoog, 1962) (Duchefa, Haarlem, Netherlands), pH 5.8 for 4 days at 30°C in the dark. Subsequently the seedlings were transferred to 96-well tip boxes (48 plants per box) and grown at 28°C, 16h light/8h dark, in half strength Hoagland solution, pH 5.8. The solution was refreshed daily. After nine days in the 96-well tip boxes, the stress was applied to 13-day old seedlings. Stressed and non-treated samples were collected at 1, 3, 6, 10, 24, and 48 h after stress application. Shoots and roots were separated, frozen in liquid N_2_ and stored at −80°C until further analysis. At least three biological replications were performed for each experiment. Rice seedlings were treated with the following abiotic stress factors: salt (150 mM), drought and plant hormones (ABA, MeJA, SA) (100 µM). Drought stress was performed by air drying the roots.

### Biotic Stress Experiments

For the biotic stress experiments sterilized seeds were germinated on MS medium, pH 5.8 supplemented with 8 g/L agar and 112 mg/L B5 vitamin (Duchefa) in a plant incubator (28°C, complete darkness, 70–75% relative humidity). Unless mentioned otherwise the seedlings were grown in perforated plastic trays (22 x 15 x 6 cm) containing autoclaved potting soil. Plants were fertilized weekly with iron solution (0.2% iron sulfate and 0.1% ammonium sulfate). The plants were kept in the greenhouse at 28°C under 16h/8h photoperiod and a relative humidity of 70 to 75% until treatment with pathogens.

Rice blast fungus (*Magnaporthe oryzae* strain VT5M1) was grown at 28°C on half-strength oatmeal agar (Difco, New Jersey, USA). Seven-day-old mycelium was placed onto the medium under blue light (mix of Philips TLD 18W/08 and Philips TLD 18W/33) for seven days to induce sporulation. At the five-leaf stage (4–5 weeks old) rice plants were inoculated with a final concentration of 4 x 10^4^ spores per milliliter in 0.5% gelatin (type B bovine skin) and mock inoculated with a solution of 0.5% gelatin by spraying until drain off. Inoculated plants were placed in a dew chamber at 28 ± 2°C under 16/8 h photoperiod and a relative humidity of more than 90% for 24 h, and then transferred to the growth chamber for further disease development for four days under the same growth conditions. Experiments were performed in three biological replicates. The fourth leaf of each plant was collected for RNA extraction at four days after inoculation. Three leaves were pooled as one biological replicate.

Rice bacterial blight (*Xanthomonas oryzae pv. oryzae*, Philippine race 6, strain PXO99) was grown on sugar peptone agar in the incubator at 28°C in the dark for 3 days. Afterwards, a single bacterial colony was inoculated into 30 mL sugar peptone broth and incubated overnight on an orbital shaker with 130 rpm at 28°C. The fully expanded and lengthy leaves of four-week-old plants were inoculated with *Xanthomonas* bacterial suspension (OD_600_ was 0.8) using the leaf clipping method as described by Kauffman et al. (1973). Mock infected plants were treated with sterile water instead of bacterial solution. The *Xanthomonas* infected and mock infected plants were allowed to grow for 2 weeks in a quarantined greenhouse set at 30°C under 16h/8h photoperiod with a relative humidity of 75%. Experiments were performed in three biological replicates. Inoculated and mock plants were collected two weeks after inoculation for RNA extraction. Twelve plants were pooled as one biological replicate.

Nematode (*Meloidogyne graminicola*) infection experiments were performed using rice seedlings grown in polyvinyl-chloride tubes containing a mixture of fine sand and synthetic absorbent polymer substrate (AquaperlaR, DCM, Belgium). Each tube contained two seedlings. The plants were grown in the plant growth room at 28°C, and supplemented with 15 mL Hoagland solution (pH 5.8) for each tube twice a week and 15 mL tap water for each tube once a week. The nematodes were cultured on *Echinochloa crus-galli* grown on potting soil. Second stage juveniles of the nematodes were extracted from 3-month-old infected roots. Fifteen-day-old rice plants were inoculated with 350 second stage juveniles or mock inoculated with autoclaved water. Both inoculated and mock plants were collected two weeks after inoculation for RNA extraction. The experiment was performed in three biological replicates, 5 plants were pooled as one biological replicate.

Insect infestation assays were performed with the brown planthopper (*Nilaparvata lugens)*. The insect colony was maintained on 4 to 8 weeks old rice plants in a growth chamber at 28°C. Low infestation was achieved by incubating 36 third instar nymphs per pot of 12 rice plants, whereas for the high infestation 100 third instar nymphs were added per pot of 12 rice plants. In the low infestation experiment, shoots of 10 infested and mock plants were collected at 5 and 13-day-after infestation for RNA extraction and pooled as one biological replicate. In the high infestation experiment, shoots of 10 infested and mock plants were collected at 3, 6 and 9 day-after infestation for RNA extraction and pooled as one biological replicate. Both infestation experiments were performed in three biological replicates.

*Pythium graminicola* (strain PB 912 132) infection experiments were done with rice seedlings grown *in vitro*. Therefore, six surface sterilized seeds were placed (2 cm apart from each other) and germinated on Gamborg B5 (Duchefa) medium supplemented with 8 g/L agar in square Petri dishes (120 × 120 mm). Plates were arranged in upstanding position (60°C angle) in the plant growth room at 28°C. Plants were grown in the dark for 3 days followed by growth in shielded light by covering the plates partially with aluminum foil on the root side. PB 912 132 was maintained on Potato Dextrose Agar medium at 28°C in the dark. Seven-day-old seedlings were inoculated by placing three mycelial plugs (5 mm in diameter) taken from the edge of a three-day-old *P. graminicola* mycelium between the roots. In mock plates, Potato Dextrose Agar medium plugs without mycelium were placed between the seedlings. Inoculated and mock plants were collected for RNA extraction 3 days after inoculation. The experiment was performed with three biological replicates, at least 12 plants were pooled as one biological replicate.

### Real-Time Quantitative PCR

The plant material was ground in liquid nitrogen and total RNA was isolated using the Spectrum™ Plant Total RNA Kit (Sigma-Aldrich, Saint Louis, USA) according to the manufacturer’s instructions. Samples were treated with RNase-free DNase I enzyme (DNase kit, Fermentas, St. Leon-Roth, Germany) to remove residual genomic DNA. Reverse transcription reactions were performed with Maxima Reverse Transcriptase Kit (Thermo Fisher Scientific, Waltham, USA) using 0.5 µg RNA and the following PCR conditions: 10 min at 25°C, 20 min at 55°C and 5 min at 85°C. qRT-PCRs were performed using SYBR Green I Mix (Bio-Rad) and following conditions: 10 min initial denaturation step at 95°C, 42 cycles (15 s at 95°C, 25 s at 60°C, 20 s at 72°C) and detected with Bio-Rad CFX Connect™ Real-Time PCR Detection System. The expression of the genes was normalized against three reference genes: *EXP* (LOC_Os03g27010.1), *EIF5C* (LOC_Os011g21990.1), and *EXP Narsai* (LOC_Os07g02340.1) (Narsai et al., 2010; Kyndt et al., 2012; Al Atalah et al., 2014). The stability of the reference genes was checked by the qbase+ software (Biogazelle, Zwijnaarde, Belgium). The software addresses minimal acceptable reference target stability by defining a threshold value for two indicators of expression stability: the geNorm expression stability value of the reference gene (M ˂ 1) and the coefficient of variation of the normalized reference gene relative quantities (CV ˂ 0.5). If a reference gene was not stabile between samples, it was excluded from the qPCR analyses. The three references genes were stabile for all the qPCR analyses, except for the SA treatment where EIF5C reference was excluded and the qPCR analyses were performed with the two remaining reference genes. All primers used for qPCR are described in **Supplementary Table 1** and control experiments are shown in **Supplementary Figure 1**. Data analysis was performed using the Bio-Rad CFX Manager software.

### Vector Construction, Generation of GUS Lines and GUS Staining

Constructs used in the GUS reporter system contained the 2-kb promoter region upstream of the translation start of each OsEUL gene. All primers used for the construction of the GUS lines are described in **Supplementary Table 2**. The promoter fragment was inserted between the KpnI and XhoI site of the pENTL4R1 plasmid by classical cloning. The transcriptional fusion of the promoter fragment with the *GUS* coding sequence was obtained by Multi Gateway Cloning (Thermo Fisher Scientific) using the entry vectors pENTL4R1, pEN-L1-S-L2 and the destination vector pBb7m24GW (obtained from Plant Systems Biology, Vlaams Instituut voor Biotechnologie, Ghent, Belgium). The expression clones were introduced into the *A. tumefaciens* strain EHA 105 by electroporation. These strains were used to transform *O. sativa* cv. Nipponbare and create promotor::GUS lines.

For the establishment of an *in vitro* callus culture for rice transformation, surface sterilized rice seeds were inoculated on callus induction medium (N6 salts and vitamins, 30 g/L sucrose, 1 g/L casein hydrolysate, 1.5 g/L L-proline, 2 mg/L 2,4 dichlorophenoxyacetic acid and 0.8% agarose; pH 5.8). Actively proliferating pre-cultured callus material was washed with liquid infection medium [MS salts (Murashige and Skoog, 1962), 68.5 g/L sucrose, 36 g/L glucose, 1 g/L casein hydrolysate, 0.87 g/L L-glutamine, 0.26 g/L L-aspartic acid, 0.17 g/L L-arginine, and 7.5 mg/L glycine; pH 5.2] and subsequently inoculated in 35 mL of *Agrobacterium* suspended in infection medium at an OD of 1.2). After 10 min, the callus material was removed from the *Agrobacterium* suspension and incubated for 4 days at 26 ± 1°C under complete darkness on sterile Whatman filter paper placed on R2–COMAS medium [MS salts, B5 Vitamins (Gamborg et al., 1968), 20 g/L sucrose, 10 g/L glucose, 1 g/L casein hydrolysate, 2 mg/L 2,4-D, 100 µM acetosyringone, and 0.8% agarose SPI; pH 5.2]. Following co-cultivation, calli were washed with sterile distilled water containing 500 mg/L timentin (Duchefa) blotted dry on sterile Whatman filter paper and subsequently placed on selection medium (CIM containing 500 mg/L timentin and 50 mg/L phosphinothricin (Duchefa). The calli were passed through 2 selection cycles of 15 days each. At the end of selection, the surviving and actively proliferating calli were inoculated on pre-regeneration medium (N6 salts and vitamins, 30 g/L sucrose, 1 g/L casein hydrolysate, 1.5 g/L L-proline, 1 mg/L 2,4-D, and 0.8% agarose; pH 5.8) supplemented with 300 mg/L timentin and 30 mg/L phosphinothricin for 4 weeks. Well proliferated calli were then selected and inoculated onto regeneration medium (0.5 x MS salts and modified vitamins (Duchefa), 20 g/L sucrose, 1 g/L casein hydrolysate, 40 µM copper sulfate, 0.87 g/L L-glutamine, 0.26 g/L L-aspartic acid, 0.17 g/L L-arginine, and 7.5 mg/L glycine, 1 mg/L zeatin riboside, and 0.8% agarose; pH 5.8) and incubated for 6 weeks. After 6 weeks, induced shoots (2-3 cm) were individually excised and transferred onto rooting medium (0.5x MS basal salts, 0.5x B5 vitamins, 10 g/L sucrose, 0.5g/L MES, and 0.8% agar; pH 5.8). After 3–4 weeks, well rooted plantlets were transplanted to soil and grown in a growth room at 28°C. The (T_0_) plants were examined on genomic level for the GUS gene and allowed to undergo self-pollination. T1 and T2 seeds were used for the experiments.

Two independent reporter lines were tested for each OsEUL gene. The GUS assay was performed according to Jefferson (1987). One week-old plants were incubated for 30 min in 90% acetone at 4°C followed by washing with phosphate buffer (0.1 M NaH_2_PO_4_.2H_2_O and 0.1 M Na_2_HPO_4_; pH 7.2). After 30 min treatment with GUS pre-incubation buffer (0.1 M phosphate buffer, 0.5 mM potassium-ferricyanide and 0.5 mM potassium-ferrocyanide) staining was performed overnight at 37°C in GUS staining buffer (2 mM X-gluc added to the preincubation buffer). After staining the plants were washed and preserved in 70% ethanol at 4°C. Microscopic slides were made with lactic acid (90%) and analyzed under the Widefield microscope Nikon eclipse Ti (Nikon instruments, Badhoevedorp, Netherlands) using a 5x dry objective lens.

### *Nicotiana benthamiana* Infiltration

The coding sequence for each OsEUL sequence was cloned after the 35S promoter in the expression vectors pK7WFG2 and pK7WGF2 to create the OsEUL-EGFP and EGFP-OsEUL constructs, respectively. The cloning started by adding attB sites to the coding sequence by PCR followed by BP reaction into pDONR221 plasmid to end with the destination vector after LR reaction. All primers used for the construction of the vectors are described in **Supplementary Table 3**. Four week old plants were transiently transformed with *Agrobacterium* strain LBA4044. The culture was inoculated in YEB medium with spectinomycin (50 µg/mL) at 28°C until an OD_600_ of 0.8 was reached. Afterwards the cells were washed twice with infiltration buffer (50 mM MES, 2mM Na_2_HPO_4_, and 0.5% glucose, pH 5.6) and finally re-suspended in infiltration buffer containing 100 µM acetosyringone. The *Agrobacterium* solution was injected in the abaxial epidermal cells of the tobacco leaves. Two days after injection, the infiltrated leaf area was analyzed under the Nikon A1R confocal laser scanning microscope (Nikon instruments) mounted on a Nikon Ti-E inverted epifluorescence body with an S Plan Fluor ELWD air objective lens (NA 0.60) or a 60_ Plan APO VC water immersion lens (NA 1.20) for 4′,6-diamidino-2-phenylindole (DAPI) and propidium iodide (PI) treated slides respectively. The tobacco leaves were infiltrated with DAPI (0.01 mg/mL) or PI (0.01 mg/mL), before making the microscopic slides of the tobacco leaves in water. EGFP was excited with a 488 nm argon ion laser, PI was excited with a 560 nm laser and DAPI was excited with a 404 nm laser. Emission filters were 500–550 nm for EGFP, 570–620 nm for PI and 425–475 nm for DAPI. Image analysis and assessment of co-localization were performed in ImageJ (Schindelin et al., 2012). Plasmolysis experiments were performed by incubating leaf samples in 2 M salt solution and analysis under the microscope (Pitzschke et al., 2014).

## Results

### Tissue Specific Expression of the OsEUL Genes

**Figure 1:**
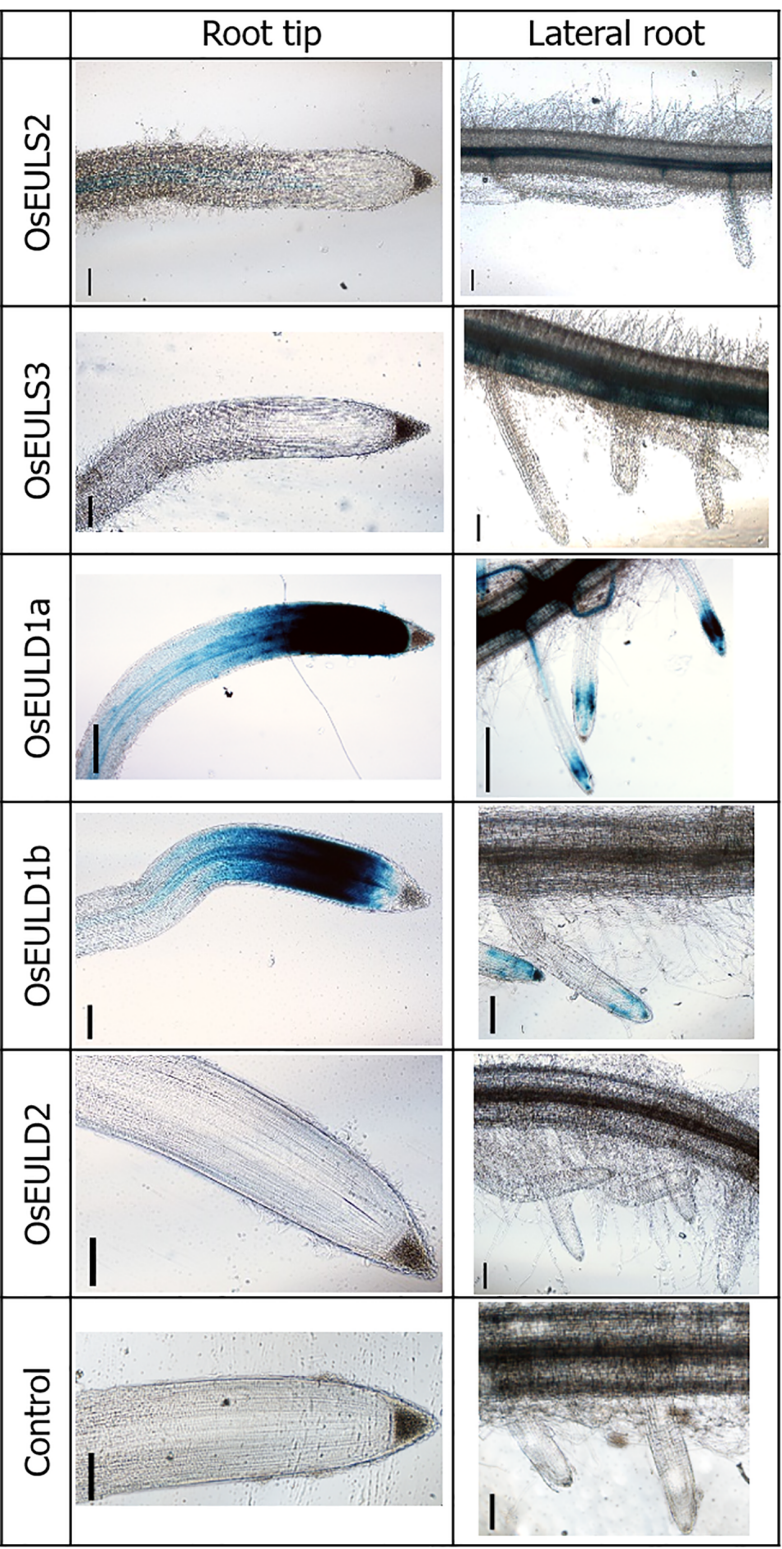
OsEUL gene patterning in rice roots indicated by promoter-driven GUS expression. Scale bar represents 0.1 mm.

To investigate the tissue-specific expression of OsEULs, GUS reporter lines were constructed. To exclude positional effects due to insertion of the sequence in the rice genome, two independent reporter lines were tested for each OsEUL gene construct. GUS gene expression driven by the promoter of each OsEUL results in a blue histochemical staining pattern representing the tissue dependent expression for each EUL (**Figure 1**).

GUS staining in the roots yielded very different results for the different promoter lines. GUS staining was only observed in the tips of the main root as well as in the lateral roots of reporter lines for *OsEULD1a* and *OsEULD1b*. The meristematic tissue of the root tip was stained but no staining was apparent in the columella. Strong staining was observed in the vascular system for the reporter lines *OsEULS3* and *OsEULD1a* whereas the *OsEULS2* and *OsEULD2* reporter lines yielded faint to no staining. Only for the *OsEULS3* promoter lines the staining intensity in the vascular system increased in more mature tissues, with intense staining in the root zones with well-developed lateral roots, and fading towards the root tip, which is completely devoid of staining (**Figure 1**).

**Figure 2:**
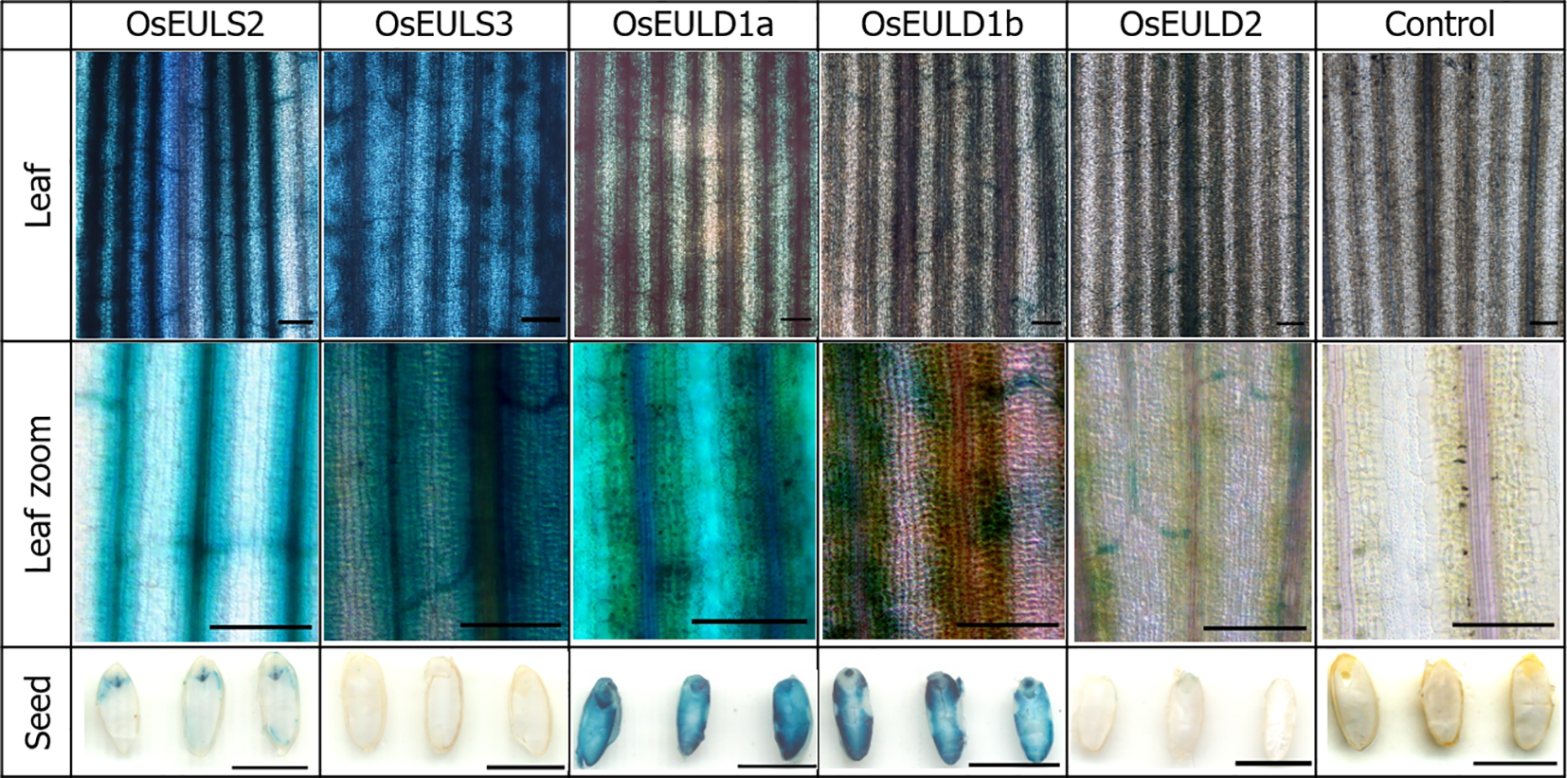
OsEUL gene patterning in rice tissues indicated by promoter-driven GUS expression for shoots and seeds. Scale bar represents 0.1 mm, except for scale bars on pictures with seeds, which represent 5 mm.

GUS staining in the leaf was observed in the longitudinal and transverse veins. A much stronger coloring was observed for the promoter constructs for the S-type EULs and *OsEULD1a* compared to the other D-type EULs. Especially the reporter lines for *OsEULD2* show a very faint signal (**Figure 2**).

Longitudinal sections of the seeds showed clear GUS staining in the embryos of the reporter lines for *OsEULS2*, *OsEULD1a*, and *OsEULD1b*. The GUS lines for *OsEULD1a* and *OsEULD1b* also showed staining outside the embryo, in the endosperm. The most explicit staining forms a ring following the starchy endosperm. The pericarp, outer layer of the rice seed, is not stained suggesting that the staining observed in the seeds for *OsEULD1a* and *OsEULD1b* is in the aleurone layer between the pericarp and starchy endosperm (**Figure 2**).

### Relative Expression Profile of the OsEULs in Shoot and Root

**Figure 3:**
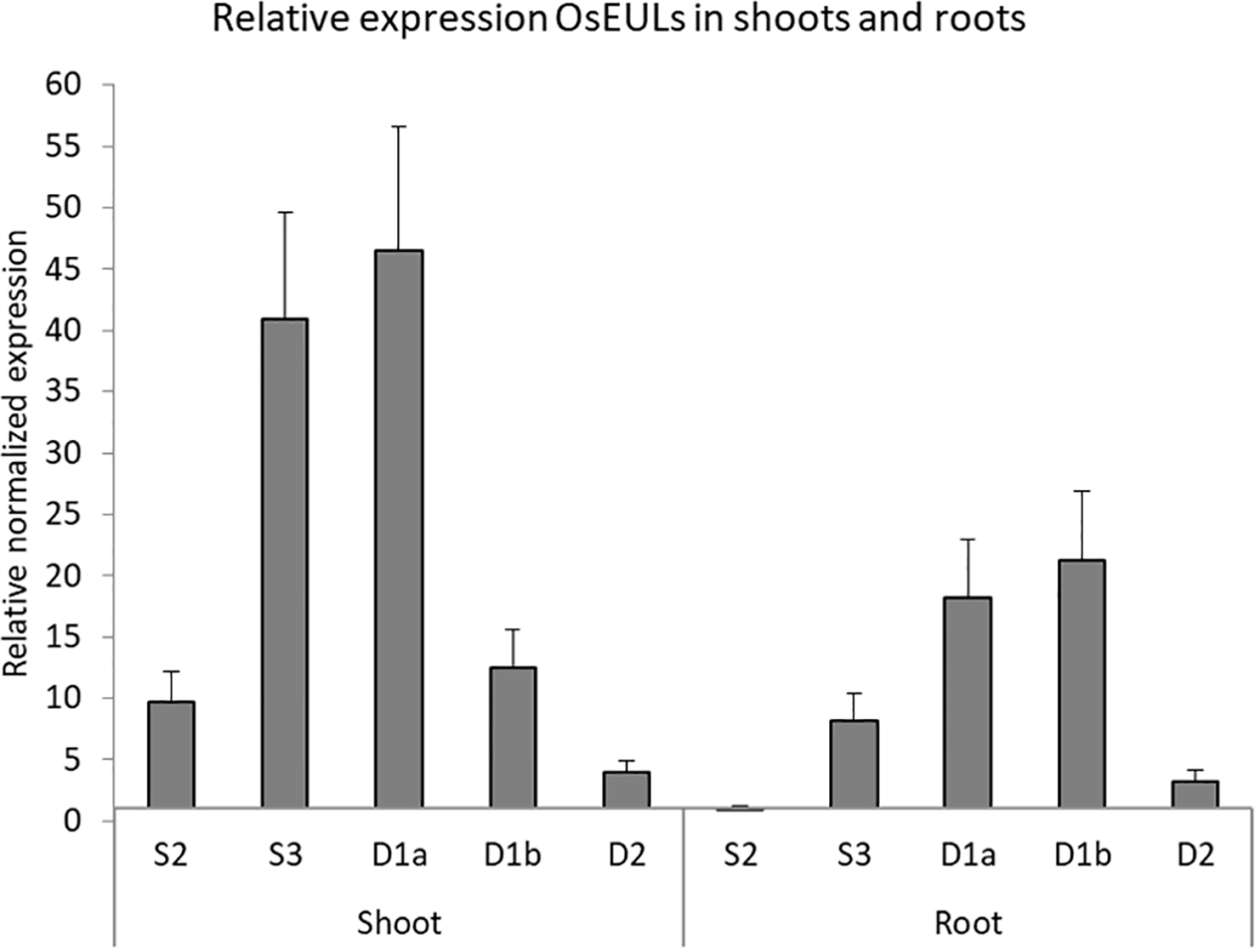
Expression profile for OsEULs in shoots and roots of 13-day old non-stressed rice seedlings. The normalized transcript levels for all EUL genes are represented relative to *OsEULS2* expression in roots. The data represent ten independent biological replicates, error bars indicate standard errors.

qPCR data allowed to compare and quantify the relative expression between the EULs in roots and shoots of rice plants grown under normal (non-stressed) growth conditions (**Figure 3**). *OsEULS3*, *OsEULD1a* show the highest expression in shoot tissue, whereas *OsEULS3*, *OsEULD1a*, and *OsEULD1b* are expressed in roots. *OsEULD1b* is the only EUL that shows higher expression in roots compared to shoots. *OsEULS2* is always present in low concentrations, but gene expression is higher in shoots compared to roots. *OsEULD2* expression levels were very low in shoots and roots under non-stress conditions.

### OsEUL Expression in Response to Abiotic Stresses and Hormonal Treatment

The expression of OsEULs was analyzed in 13-day old rice seedlings subjected to a range of plant hormones, abiotic and biotic stress conditions, including the most damaging and economically important stress factors influencing rice growth and yield. The plants were treated with the stressor for 1, 3, 6, 10, 24, and 48 h, and compared to non-treated plants harvested at the same time point.

**Figure 4:**
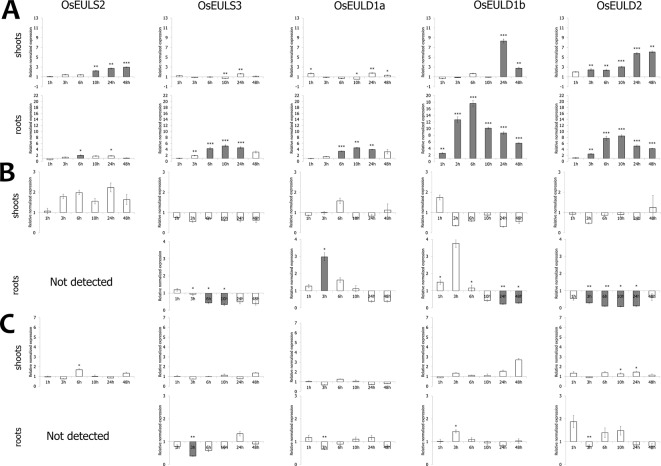
Transcript levels for OsEUL genes after different hormone treatments, including ABA **(A)**, MeJA **(B)** and SA **(C)**. Normalized expression levels, relative to the non-treated plants, set to 1, are shown for different time points. The mean values of RT-qPCR for three independent biological replicates were normalized to three reference genes and error bars indicate standard errors. Dark-gray bars represent statistically significant up-regulation of at least two-fold compared to non-treated plants and light-gray bars represent statistically significant down-regulation (^*^p < 0.05, ^**^p < 0.01, ^***^p < 0.001).

**Figure 5:**
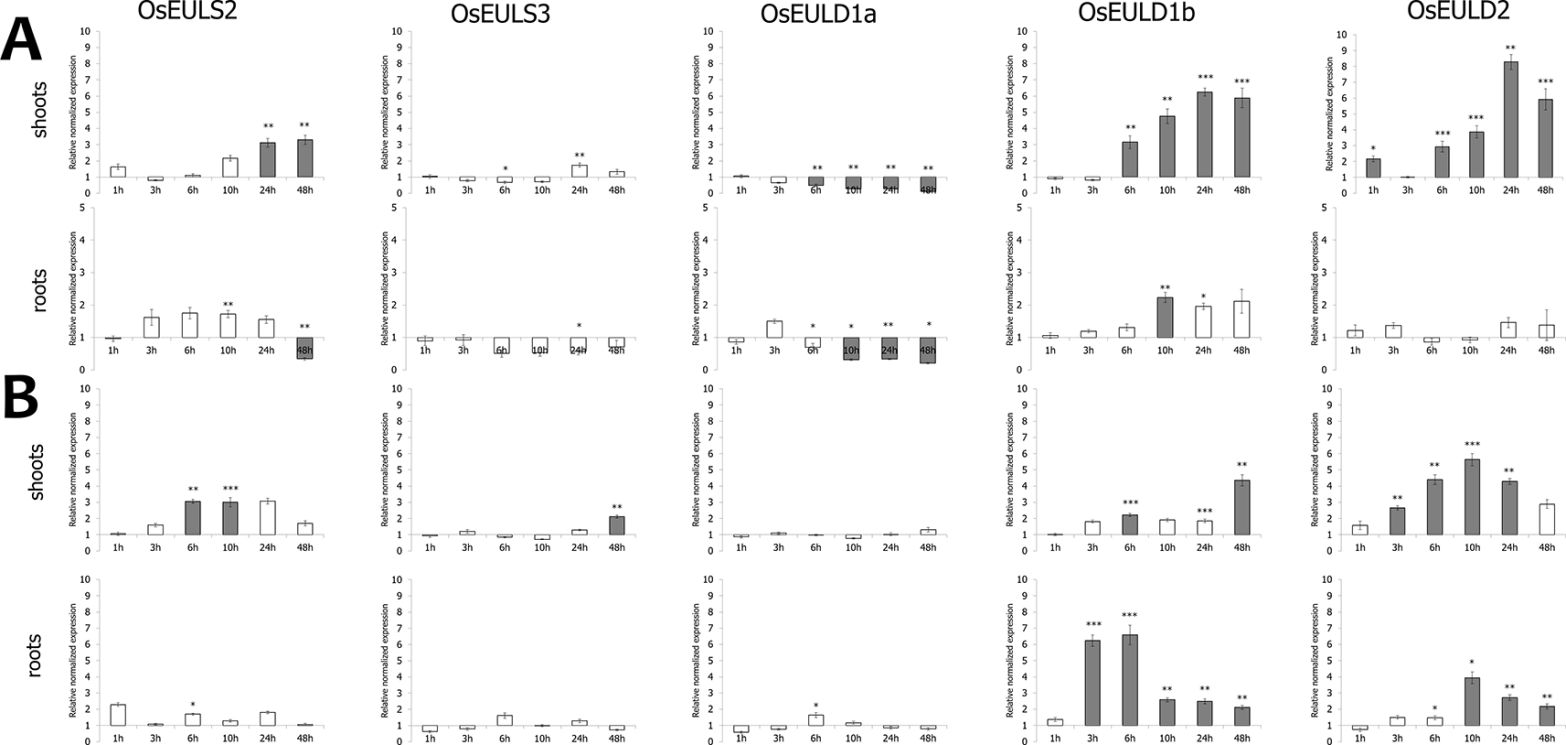
Transcript levels for OsEUL genes after drought **(A)** and salt stress **(B)**. Normalized expression levels, relative to the non-treated plants (set to 1), are shown for different time points. The mean values of RT-qPCR for three independent biological replicates were normalized to three reference genes and error bars indicate standard errors. Dark-gray bars represent statistically significant up-regulation of at least two-fold compared to non-treated plants and light-gray bars represent statistically significant down-regulation (^*^p < 0.05, ^**^p < 0.01, ^***^p < 0.001).

Of all abiotic and hormonal treatments tested, ABA treatment, drought and salt application showed the most prominent effect on EUL gene expression (**Figures 4** and **5**). ABA was the only stress factor which provoked a significant upregulation for all OsEUL transcripts, predominantly in roots and to a lesser extent in the shoots. The highest expression levels were observed for *OsEULD1b* (approximately 18-fold significant upregulation in roots after 6 h and 10-fold after 10 h treatment) and *OsEULD2* (approximately 8-fold significant upregulation in roots after 6 h and 10 h). Transcript levels for *OsEULD1a* and *OsEULS3* were also significantly upregulated in roots ranging from 3-5 to maximum 4-6-fold at 6-24 h after stress application (**Figure 4**). The highest levels of upregulation in the shoots were observed for *OsEULD1b* at 24 h and *OsEULD2* at 48 h after the treatment reaching maximum levels of 8 and 6-fold. *OsEULS2* expression was only weakly affected after ABA treatment (**Figure 4**).

The hormone treatments with MeJA and SA did not show a prominent up regulation on the transcript levels for any of the OsEUL genes. In contrast, MeJA treatments resulted in clear downregulation of most OsEUL levels. A slight upregulation was only observed in the early time points for *OsEULD1a* and *OsEULD1b* in the roots after MeJA treatment followed by statistically significant downregulation in the late time points for all D-type OsEUL genes (**Figure 4**). SA treatment yielded a significant down regulation of OsEULS3 transcript levels in roots (**Figure 4**). The *OsEULS2* transcript was not detected in the roots for both hormone treatments (**Figure 4**), due to the low expression level of *OsEULS2* in roots (**Figure 3**).

Drought stress provoked statistically significant upregulation of transcript levels for *OsEULD1b*, *OsEULD2* and *OsEULS2* in the shoot tissue (**Figure 5**). *OsEULD1a* transcripts were significantly downregulated in root and shoot tissue as a result of drought treatment, especially in the later time points. Similar to ABA treatment, the highest levels for *OsEULD1b* and *OsEULD2* were observed for the 24h time point with a gradual increase in the transcript levels reaching up to 6-8-fold change. A similar trend was observed for *OsEULS2* with expression levels that were 3-fold higher after 24 h of drought stress compared to the untreated plants (**Figure 5**). Drought treatment yielded a significant up regulation of *OsEUL1b* transcript levels in roots of approximately 2-fold after 10h (**Figure 5**).

Salt treatment triggered upregulation for *OsEULS2*, *OsEULD1b* and *OsEULD2* transcripts starting from the 6 h time point and after 48 h for *OsEULS3* in the shoots. In roots, transcript levels for *OsEULD1b* peaked after 6 h gradually decreased afterwards. *OsEULD2* transcript levels are upregulated after 10 h salt treatment in the root (**Figure 5**).

### OsEUL Expression in Response to Biotic Stresses

Transcript levels for OsEULs have been quantified after exposure of rice seedlings to some major pathogens, in particular *P. graminicola* (oomycete), *M. graminicola* (nematode), *N. lugens* (insect) and *M. oryzae* (fungus) and *X. oryzae* (bacteria).

**Figure 6:**
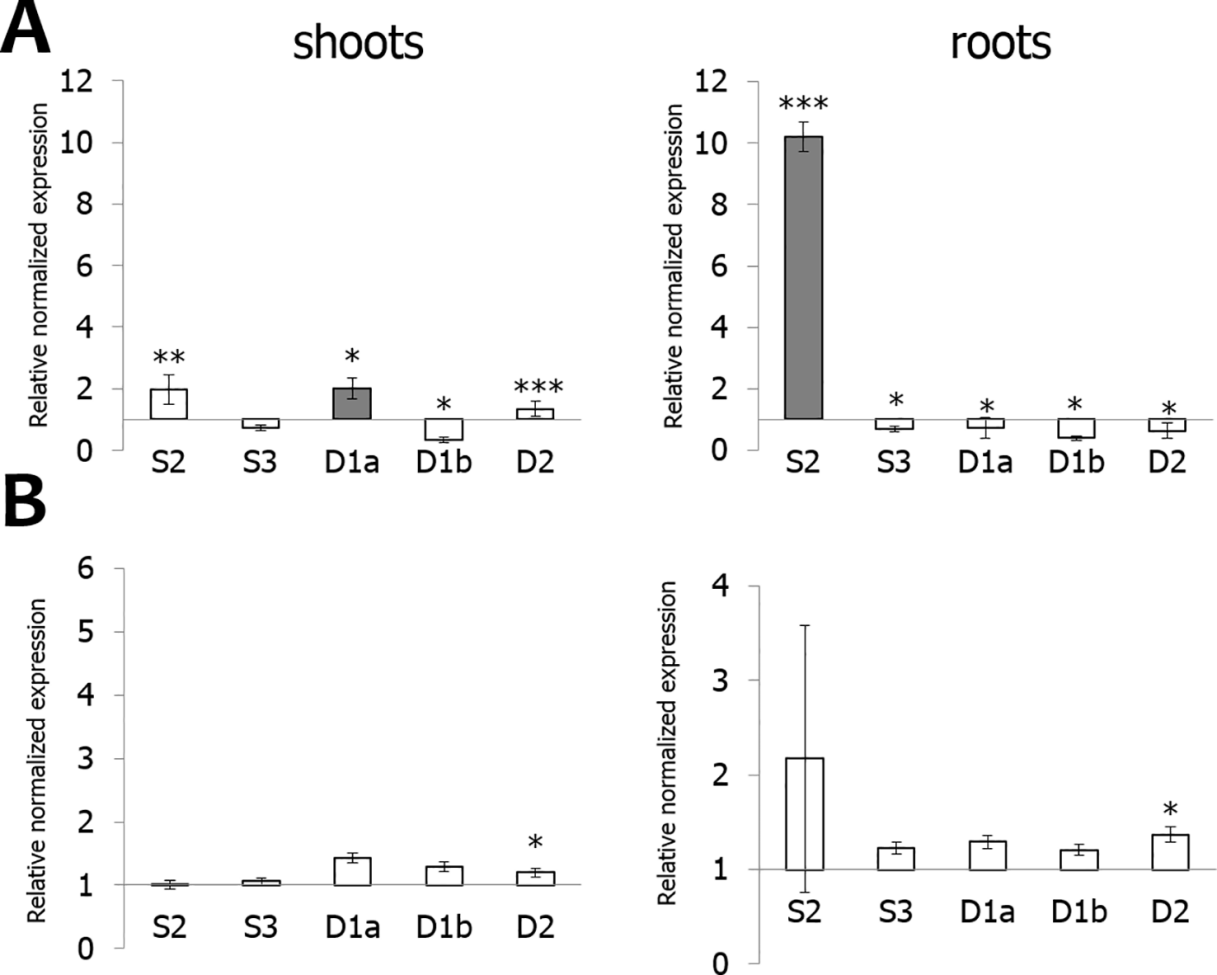
Transcript levels for OsEUL genes after infection with *Pythium graminicola* **(A)** and *Meloidogyne graminicola* **(B)**. RT-qPCR analyses were performed on shoot and root material collected at 3 dpi after *P. graminicola* and 14 dpi after *M. graminicola* infection. Normalized expression levels, relative to the non-treated plants (set to 1), are indicated. The mean values of RT-qPCR from three independent biological replicates were normalized to three reference genes. Error bars indicate standard errors. Dark-gray bars represent statistically significant up-regulation of at least two-fold compared to non-treated plants and light-gray bars represent statistically significant down-regulation (^*^p < 0.05, ^**^p < 0.01, ^***^p < 0.001).

Infection of rice seedlings with typical root pathogens affected OsEUL expression mainly in roots. At 3 dpi, the transcript levels for *OsEULS3* and all D-type OsEULs were decreased in roots of plants infected with the oomycete *P. graminicola*, whereas *OsEULS2* expression was 10-fold upregulated. In rice shoots, significant upregulation was recorded for *OsEULD1a*, while *OsEULD1b* expression was slightly but significantly downregulated after *Pythium* infestation (**Figure 6**). Infection with the nematode, *M. graminicola*, only results in a 2-fold upregulation of *OsEULS2* in the root. No changes in expression above two-fold were observed in shoot tissue after nematode infection (**Figure 6**).

**Figure 7:**
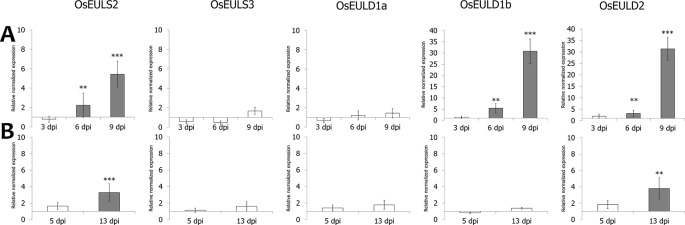
Transcript levels for OsEUL genes after infestation with *Nilaparvata lugens*. RT-qPCR analyses were performed on leaf material collected at indicated dpi after high **(A)** and low **(B)** infestation with *N. lugens*. Normalized expression levels, relative to the non-treated plants (set to 1), are indicated. The mean values of RT-qPCR from three independent biological replicates were normalized to three reference genes. Error bars indicate standard errors Dark-gray bars represent statistically significant up-regulation of at least two-fold compared to non-treated plants and light-gray bars represent statistically significant down-regulation. (*p < 0.05, **p < 0.01, ***p < 0.001). The statistics were performed (*p < 0.05, **p < 0.01, ***p < 0.001). The data was non-significant or was significant for **p < 0.01, ***p < 0.001, but none of the data was in between meaning *p < 0.05.

OsEUL expression was analyzed in shoot material of rice plants infested with the brown planthopper, *N. lugens*. A comparative analysis was made between plants subjected to low and high insect infestation. As shown in **Figure 7**, infestation of rice plants with a high number of nymphs yielded higher response from the plant compared to low infestation levels. Transcript levels for *OsEULD2*, *OsEULD1b* and *OsEULS2* are significantly upregulated starting from 6 dpi after high infestation with *N. lugens*, reaching approximately 31-, 30-, 5-fold upregulation at 9 dpi (**Figure 7**). In plants subjected to low levels of infestation with *N. lugens*, transcript levels for *OsEULD2* and *OsEULS2* were 4-fold up at 13 dpi (**Figure 7**). *N. lugens* does not alter the expression of the *OsEULS3* and *OsEULD1a* genes. No significant differences in expression were observed for the early time points after high and low infestation (3 dpi and 5 dpi, respectively), suggesting a late response of the plant (**Figure 7**).

**Figure 8:**
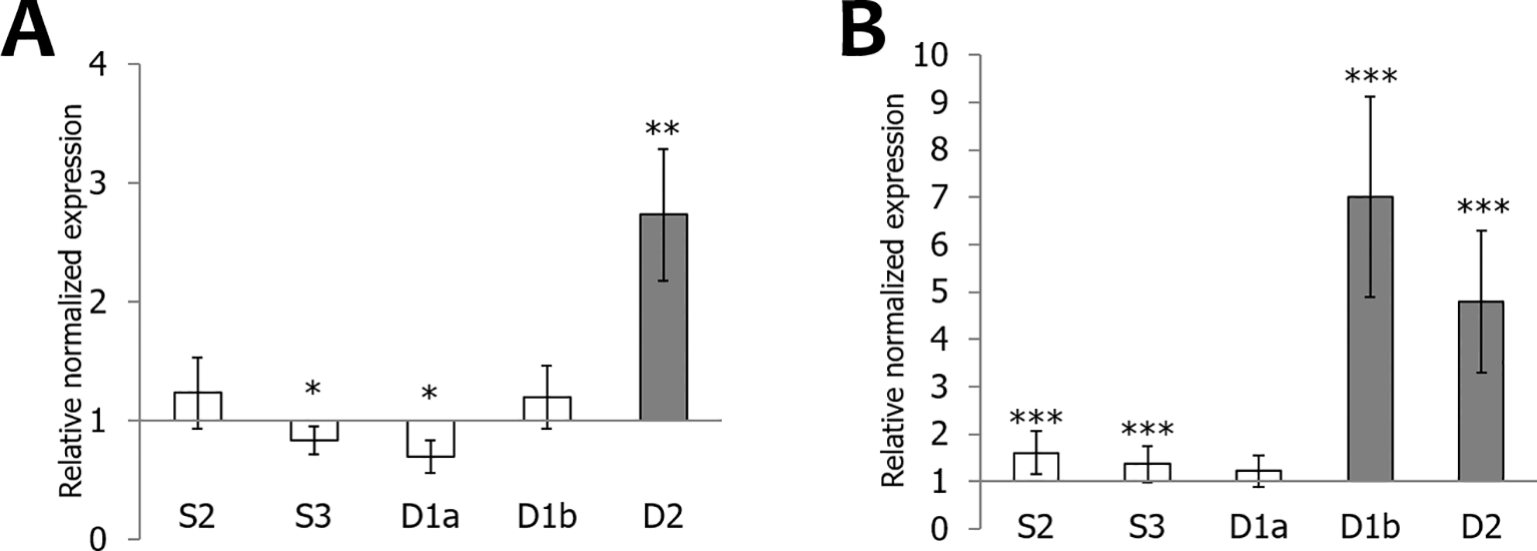
Transcript levels for OsEUL genes after infection with *Magnaporthe oryzae* **(A)** and *Xanthomonas oryzae* **(B)**. RT-qPCR analyses were performed on leaf material collected at 3 dpi after infection. Normalized expression levels, relative to the non-treated plants (set to 1), are indicated. The mean values of RT-qPCR from three independent biological replicates were normalized to three reference genes. Error bars indicate standard errors Dark-gray bars represent statistically significant up-regulation of at least two-fold compared to non-treated plants and light-gray bars represent statistically significant down-regulation (^*^p < 0.05, ^**^p < 0.01, ^***^p < 0.001).

*M. oryzae* infection especially affects the transcript levels for *OsEULD2*, resulting in a significant upregulation (3-fold) at 4 dpi. In contrast, levels for *OsEULD1a* and *OsEULS3* are significantly downregulated (**Figure 8**). Bacterial infection with *X. oryzae* results in significant upregulation of the transcript levels for *OsEULD1b* and *OsEULD2* by 7- and 5- fold, respectively. Transcript levels for *OsEULS2*, *OsEULS3* and *OsEULD1a* were not altered for more than two-fold after *X. oryzae* infection (**Figure 8**).

### Cellular Localization of the OsEUL Proteins

**Figure 9:**
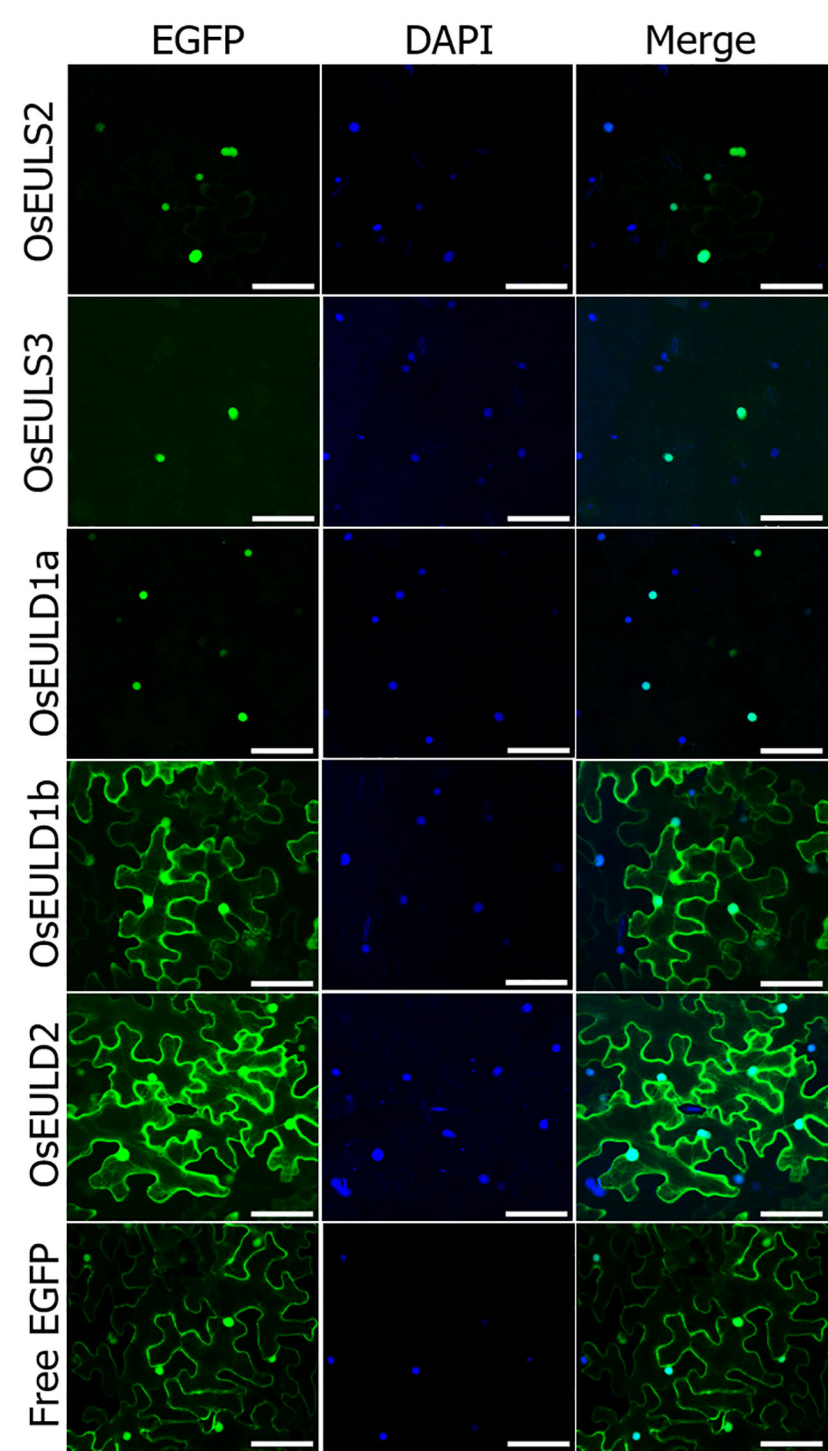
Subcellular localization of OsEUL-EGFPs and free EGFP in *Nicotiana benthamiana* lower epidermal cells. Nuclei were stained with DAPI. Co-localization between DAPI and EGFP signals was assessed in overlay pictures (Merge). Microscopy settings were identical for all pictures. Scale bars represent 80 µm.

All rice EUL genes are synthesized without a signal peptide suggesting that the OsEULs will be translated on the ribosomes in the cytoplasm and will not follow the secretory pathway. EGFP fusion constructs were made to investigate the subcellular localization for each OsEUL after transient transformation into *Nicotiana benthamiana* epidermal cells. Microscopic analysis for each OsEUL yielded fluorescence in the nucleus for the C-terminal EGFP fusion constructs (**Figure 9**). Similar localization patterns were observed for fusion proteins with a N-terminal EGFP tag. A construct expressing free EGFP was also infiltrated in tobacco leaves and served as a control. Fluorescence for free EGPF was observed in the nucleus and the cytoplasmic compartment (**Figure 9**). Co-localization experiments in which nuclei were also stained with DAPI confirmed that all OsEULs and free EGFP display expression in the nuclear compartment. Fluorescence intensity plots for DAPI and EGFP in cross sections spanning the nucleus confirmed that the EGFP fusion proteins are present in the nuclei, as shown in **Supplementary Figure 2**.

**Figure 10:**
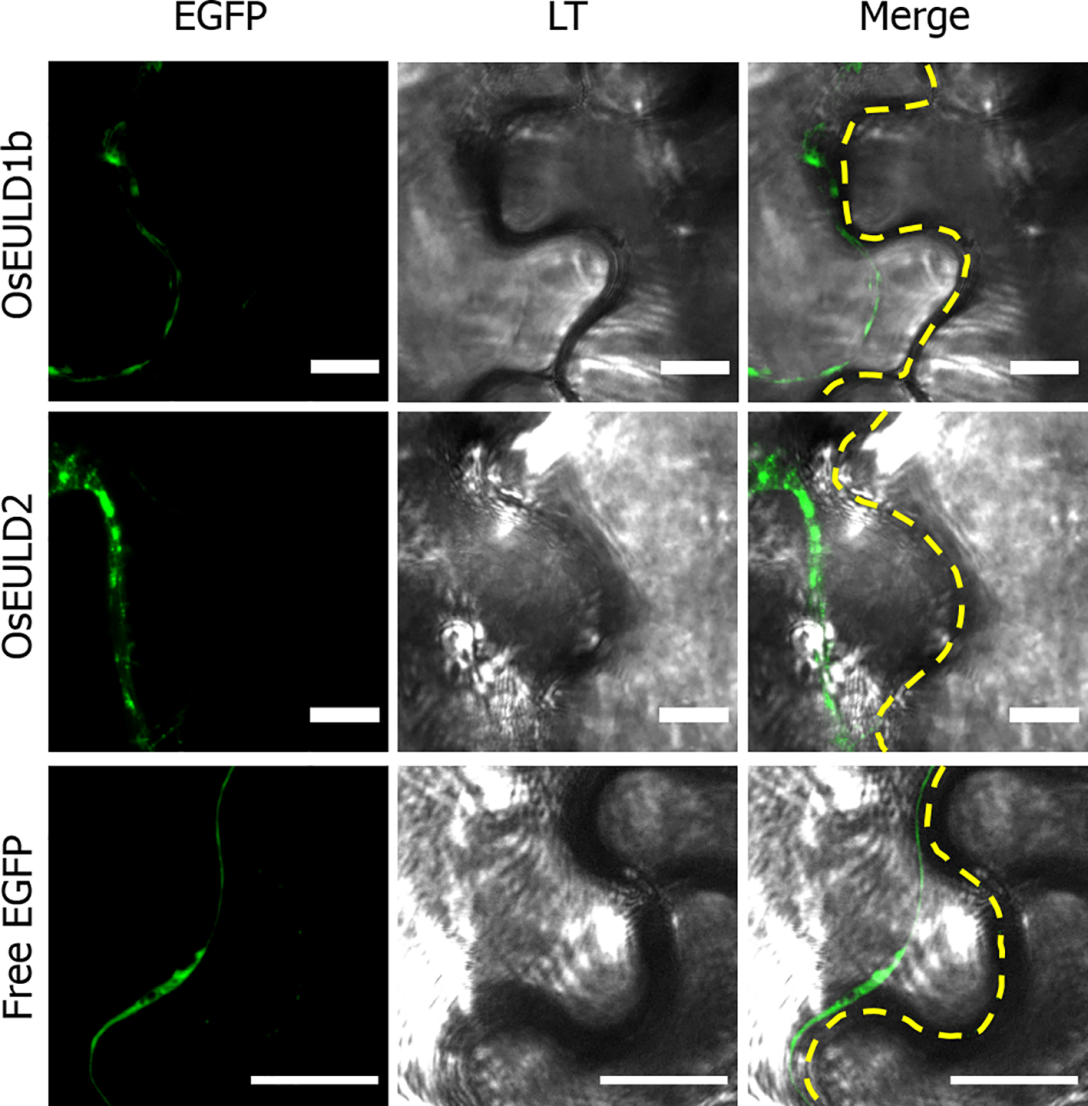
Zoom in of subcellular localization of OsEULD1b and OsEULD2 and free EGFP in plasmolyzed *Nicotiana benthamiana* lower epidermal cells at the cell border. Fluorescence signal (EGFP) from free EGFP, OsEULD1b:EGFP and OsEULD2:EGFP are presented. Light transmission (LT) microscopy showed the cell walls. Co-localization of LT and EGFP staining was assessed in overlay pictures (Merge). The dashed white line marks the cell wall. Scale bars represent 10 µm.

The OsEULD1b and OsEULD2 fusion constructs with EGFP also showed fluorescence in the cytoplasm. A plasmolysis experiment confirmed that fluorescence is present in the cytoplasm but is excluded from the cell wall (**Figure 10**). Fluorescence intensity plots for PI and EGFP cross sections spanning the cell borders of neighboring cells confirmed the fluorescence for OsEULD1b and OsEULD2 in the cytoplasm and not in the cell wall (**Supplementary Figure 3**).

To check whether the localization patterns might change in stress conditions, localization experiments were also performed for transiently transformed tobacco leaves subjected to 50 µM ABA stress for 1h. ABA was selected as the best stress condition since the expression of all OsEULs was affected by ABA treatment (**Figure 4**). The cellular localization for the OsEUL proteins did not change after ABA treatment (**Supplementary Figure 4**).

## Discussion

Rice (*O. sativa*) is one of the most important staple foods in the world and is cultivated in all habitable continents. According to the Food and Agricultural Organization of the United Nations (Katayama et al., 2019) more than 3.5 billion people depend on rice for more than 20% of their daily calories. Rice is the second most produced cereal and is a model organism for other important cereals, including maize and wheat, making it a highly important crop for agriculture and translational research (Matsumoto et al., 2005; Li et al., 2014; Muthayya et al., 2014). Unfortunately, the rice crop is highly susceptible to abiotic stresses, especially to drought and salinity. In addition, rice is prone to infection by bacteria, fungi, nematodes, oomycetes, and insects. It is generally accepted that plants use a whole array of proteins to cope with undesirable conditions (Bellande et al., 2017). Therefore, several rice proteins, among which lectins and ribosome-inactivating proteins, have been studied aiming to identify proteins that can help to create plants with better performance and stress tolerance.

The *Euonymus*-related lectins (EULs) represent a family of stress-related lectins composed of one or two EUL domains. The EUL domain is unique in that it is ubiquitous in land plants, implying that these proteins play a significant role. The rice genome contains five evolutionary related OsEUL sequences, encoding two groups of structurally diverse proteins, in particular the S-type and D-type proteins, harboring one or two EUL domains, respectively. Only limited information is available for the OsEUL proteins, probably due to the low expression levels of OsEULs in plant tissues. However, a few transcriptomic as well as proteomic studies have provided indications that OsEULs represent stress related proteins (Moons et al., 1995; Rabello et al., 2008). The aim of this study was to make a comparative analysis of the subcellular localization and tissue expression, as well as the stress responsiveness of the different OsEULs.

GUS activity was detected in the vascular system of all GUS reporter lines, but the staining pattern was different for each promotor line, suggesting that the expression of the OsEULs is tissue specific. Promoter activity for *OsEULS3*, *OsEULD1a*, and *OsEULD1b* was clear in roots as well as in shoots. Unlike all other OsEULs, *OsEULS3* never showed GUS staining in the vascular system at the root tip. *OsEULD2* has a lower basal expression compared to the other OsEULs. *OsEULS2*, *OsEULD1a*, and *OsEULD1b* are also expressed in the embryo pointing towards a potential role in primary growth. *OsEULD1a* and *OsEULD1b* are expressed in the seed and mainly in the endosperm. Interestingly, the endosperm has a function in seed dormancy, which is sensitive to the abiotic stress hormone ABA (Yan et al., 2014). Both *OsEULD1a* and *OsEULD1b* are expressed in the meristematic zone of the root tips from the main and the lateral roots. This meristematic zone is the location where plant cells undergo cell division. Root growth is achieved by cell division followed by cell elongation (Sanz et al., 2011). The study of the promoter elements in the promoter sequence of *OsEULD1b* revealed that *OsEULD1b* has ABA related elements, such as TRAB1 and OSBZ8 (De Schutter et al., 2017). OSBZ8 is reported in the roots and developing embryos, while TRAB1 shows expression in the embryo and seeds (Banerjee and Roychoudhury, 2017). These data are consistent with the results of the GUS staining.

ABA is considered as one of the key players in plant growth and abiotic stress response. For instance, NCED, an important enzyme in the ABA biosynthesis pathway, is upregulated by drought and salt resulting in higher levels of ABA (Huang et al., 2019). Low concentrations of ABA are required for primary root elongation and growth recovery in response to water deficit and salt stress (Sharp et al., 2004; Geng et al., 2013). Transcript levels for *OsEULD1b* and *OsEULD2* were significantly upregulated after salt, drought and ABA treatment. Interestingly, *OsEULD1a* were downregulated after drought treatment, which seems contradictory since *OsEULD1a* levels were also upregulated by ABA in the roots. However, plants can cope with drought by utilizing both ABA dependent and ABA independent pathways (Borah et al., 2017). Salinization and drying of the soil alter the fluxes in the vascular system. The concentration of ABA in the vascular system will increase in response to drought and salt stress. Furthermore, the ABA concentration will increase much faster in the roots than in the leaf after drought stress, which explains the late response in the shoots (Pérez-Alfocea et al., 2011). Proteomics studies for ABA treated roots reported the upregulation of OsR40C1, here referred to as OsEULD1b (Moons et al., 1995; Rabello et al., 2008). In contrast to the significant increase of OsEUL expression after treatment of ABA, MeJA treatment resulted in a decrease of EUL transcript levels whereas SA treatment did not show any major effect on OsEUL levels. Crosstalk exists between MeJA and ABA signaling in plants and many genes are commonly regulated by MeJA and ABA (Kim et al., 2018). It has been shown that MeJA can stimulate stomatal closure similar to ABA, but can also act as an antagonist to ABA in rice roots through inhibition of primary root elongation resulting in reduced root length (Savchenko et al., 2014; Liu et al., 2015).

Defense responses are modulated by the induced production of a hormonal blend and are crucial to protect plants against several plant attackers. The plant hormones ABA, MeJA and SA are important regulators of induced defense mechanisms (Vos et al., 2015). MeJA plays a critical role in rice immunity, and is necessary in the defense against the nematode *Hirschmanniella oryzae* and *M. graminicola* whereas ABA suppressed host resistance against *Magnaporthe* by inhibiting SA signaling. A recent study showed that MeJA levels are increased after foliar application of abamine (an inhibitor of ABA biosynthesis) in the roots whereas the foliar application of ABA led to MeJA suppression. ABA acts as an antagonist to MeJA biosynthesis and its signaling pathway, making the plants more susceptible to the pests (Nahar et al., 2011; Kyndt et al., 2017). Transcript levels for all EULs except for OsEULS2 were significantly downregulated in roots by the root pathogen *P. graminicola*. However, this downregulation was not observed for the root nematode *M. graminicola*, indicating that several root pathogens affect transcript levels differently. Future experiments with transgenic lines overexpressing the OsEULs will allow to investigate the role of OsEULs in rice defense.

Generally OsEUL transcript levels are upregulated more prominently after biotic stresses compared to abiotic stresses. However, it is also well known that some pathogen infections also affect hormone levels. For instance, MeJA signaling plays a vital role in plant defense against chewing insects, but makes plants more susceptible to infestation by sucking insects, such as the brown plant hopper *Nilaparvata* (Tong et al., 2012). *OsEULS2*, *OsEULD1b*, and *OsEULD2* were significantly upregulated after high infestation with the brown plant hopper. Moreover *OsEULD2* and *OsEULD1b* transcript levels were also upregulated after *Xanthomonas* infection. *OsEULD2* was significantly upregulated after *Magnaporthe* infection. These results are in agreement with a previous qPCR study using different time points which also showed the upregulation of *OsEULD1b* and *OsEULD2* after *Xanthomonas* infection and the upregulation of *OsEULD2* after disease by *Magnaporthe* (Al Atalah et al., 2014). Transcript levels for *OsEULD2* were significantly upregulated by three different rice pathogens. *OsEULD2* levels were upregulated after stress provoked by *M. oryzae, X. oryzae* and *N. lugens*, suggesting that *OsEULD2* is generally more stress responsive compared to the other OsEULs.

Our results give a complete and conclusive overview of the stress responsiveness of the OsEUL genes to single stresses. However, in nature plants are exposed to multiple stresses. Recent studies showed that combined stress is regulated by overlapping cellular signaling mechanisms that are different from studies with individual stresses (Vemanna et al., 2019). However, judging from the stress treatments performed in this study we can conclude that the EUL gene family in rice is stress responsive, and reacts to a broad range of stresses, making the OsEULs good candidate genes for creating plants with durable resistance to multiple stresses. Furthermore it is clear that multiple OsEULs in rice act differently, and probably exert complementary activities (**Tables 1** and **2**).

**Table 1:** Overview of OsEUL expression in response to abiotic and hormone treatments.

		ABA	Salt	Drought	MeJA	SA
OsEULS2	Shoot	↗	↗	↗		
OsEULS3						
OsEULD1a				↘		
OsEULD1b		↗	↗	↗		
OsEULD2		↗	↗	↗		
OsEULS2	Root	↗			n.d.	n.d.
OsEULS3		↗			↘	↘
OsEULD1a		↗		↘	↘	
OsEULD1b		↗	↗	↗	↘	
OsEULD2		↗	↗	↗	↘	

Green and red arrows indicate significant upregulation and downregulation of expression, respectively, for at least two-fold. n.d., not detected.

**Table 2:** Overview of OsEUL expression in response to biotic treatments. Green and red arrows indicate significant upregulation and downregulation of expression, respectively, for at least two-fold.

Pathogen/Insect	Tissue	OsEULS2	OsEULS3	OsEULD1a	OsEULD1b	OsEULD2
*Pythium graminicola*	Root	↗				
	Shoot			↗		
*Melidogyne graminicola*	Root					
	Shoot					
*Nilaparvata lugens*	Shoot	↗			↗	↗
*Magnaporthe oryzae*	Shoot					↗
*Xanthomonas oryzae*	Shoot				↗	↗

The rice plant contains multiple lectins, some of which are expressed in the same tissues. For instance, next to EULs, rice also expresses *Orysata*, a nucleocytoplasmic jacalin-related lectin. *Orysata* expression is enhanced after drought and salt stress as well as SA, MeJA, ABA treatment and *M. oryzae* attack (Garcia et al., 1998; De Souza Filho et al., 2003; Shinjo et al., 2011; Al Atalah et al., 2014). Similar to OsEULs, *Orysata* shows expression in roots and shoots. Overexpression of *Orysata* in rice plants was reported to improve salinity tolerance (He et al., 2017). Recently, *Orysata*, also known as “SALT”, was identified in Saltol-1, a major quantitative trait locus related to salt stress, as the major contributor to the salt stress resistance (Patishtan et al., 2018). Orysata interacts with RING finger proteins suggesting that it is regulated by the ubiquitination pathway (Van Holle and Van Damme, 2018).

The EUL sequences are synthesized without a signal peptide and therefore EULs are presumably translated on free ribosomes in the cytosol of the plant cell. Our data show that all OsEULs fused to EGFP locate to the nucleus. Moreover, the S-type EULs and OsEULD1a are detected only in the nucleus whereas OsEULD1b and OsEULD2 also reside in the cytoplasm. None of the EUL sequences contains a classical nuclear localization site (NLS). At present, it remains unclear how the OsEUL proteins are at least partially translocated from the cytosol to the nucleus. The nuclear transport could be by passive diffusion through the nuclear pore or active nuclear transport which does not rely on the presence of a classical NLS (Wang and Brattain, 2007; Yao et al., 2014). At present the role of EULs in the nucleus is not known. It is possible that OsEULs act in similar way as described for the nucleocytoplasmic lectins *Orysata* and *Nictaba*. *Nictaba* is a nucleocytoplasmic lectin from tobacco, it acts as a stress-inducible modulator of gene transcription by chromatin remodeling through binding to O-GlcNAc modified core histones (Schouppe et al., 2011; Delporte et al., 2014). Overexpression of Nictaba-like lectin genes from *Glycine max* confers tolerance toward *P. syringae* infection, aphid infestation and salt stress in transgenic *Arabidopsis* plants (Van Holle et al., 2016), suggesting that Nictaba can regulate the plant immune response.

Only few stress inducible lectins have been studied and functionally characterized. This paper highlights the tissue-specific expression, the subcellular localization and the stress inducibility of a family of closely related lectins from rice. The experimental data confirm the stress dependent regulation of the EUL lectins for a broad range of stresses, like drought and salinity, and infections by bacteria, fungi, oomycetes and insect attack. Despite the fact that OsEUL sequences show a high degree of sequence similarity they show marked differences in subcellular localization and tissue expression, as well as in their stress responsiveness, suggesting that they play a different or a complementary role in the rice plant. More research, such as the generation of rice plants overexpressing OsEULs, is needed to experimentally prove the involvement of EULs in stress signaling and plant defense in rice. Furthermore, the identification of interaction partners for OsEULs will contribute to define the physiological role and the importance of these genes in the plant.

## Data Availability Statement

All datasets generated for this study are included in the article/**Supplementary Material**.

## Author Contributions

JL, SD, MD, MT, PW, JZ, and ED outlined and designed the study. JL, SD, MD, MT, PW, JZ, IV, PW, and KS performed the experiments. JL, SD, MD, PW, and JZ analyzed and interpreted the data. JL, SD, MD, MT, and ED prepared the manuscript. GS assisted with the design and interpretation of the insect experiments. ED conceived and supervised the experiments and critically revised the manuscript. All authors have read, revised, and approved the final manuscript.

## Funding

This research was funded by the Research Council of Ghent University (project BOF15/GOA/005) and the Fund for Scientific Research–Flanders (FWO Grant G006114N).

## Conflict of Interest

The authors declare that the research was conducted in the absence of any commercial or financial relationships that could be construed as a potential conflict of interest.
