# Supplementary material for: OsEUL Lectin Gene Expression in Rice: Stress Regulation, Subcellular Localization and Tissue Specificity
Source: Front Plant Sci. 2020 Mar 2;11:185. doi: 10.3389/fpls.2020.00185 (PMC7061729; doi:10.3389/fpls.2020.00185)
Supplement: Supplementary file 1 [file DataSheet_1.pdf]

Supplementary Material

EUL gene expression in rice: stress regulation, subcellular localization and tissue specificity.

Jeroen Lambin, Sinem Demirel Asci, Malgorzata Dubiel, Mariya Tsaneva, Isabel Verbeke, Pieter Wytynck, Jeroen De Zaeytijd, Guy Smagghe, Kondeti Subramanyam and Els J. M. Van Damme*

*** Correspondence:** Prof dr Els JM Van Damme: elsjm.vandamme@ugent.be

1 Supplementary Figures and Tables

1.1 Supplementary Figures

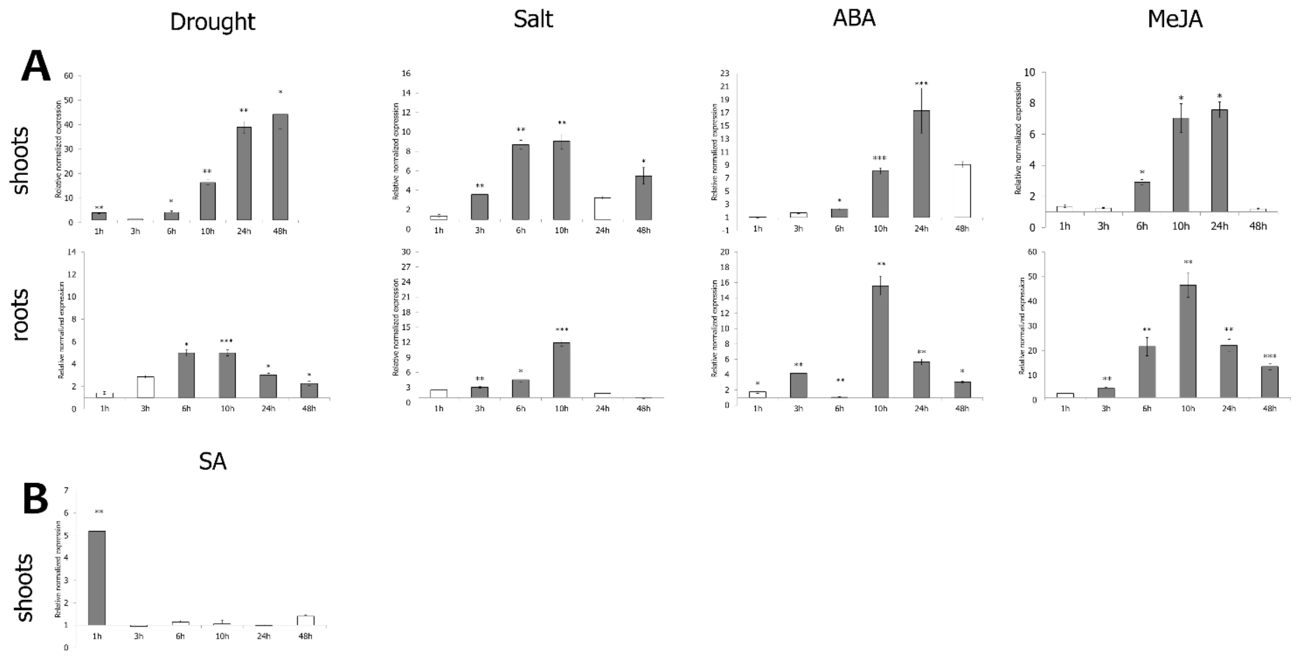

Supplementary Figure 1. Controls for the stress conditions imposed by different hormonal and abiotic stress treatments. Orysata is used as positive control for drought, salt, ABA and MeJA treatment (A). WRKY45 is used as a positive control for SA treatment (B). Normalized expression levels, relative to the control (non-treated plants, set to 1), are shown for different time points. The mean values of RT-qPCR for three independent biological replicates were normalized to three reference genes and error bars indicate standard errors. Dark-gray bars represent statistically significant up-regulation of at least two-fold compared to non-treated plants and light-gray bars represent statistically significant down-regulation ($^{*}p < 0.05$, $^{**}p < 0.01$, $^{***}p < 0.001$).

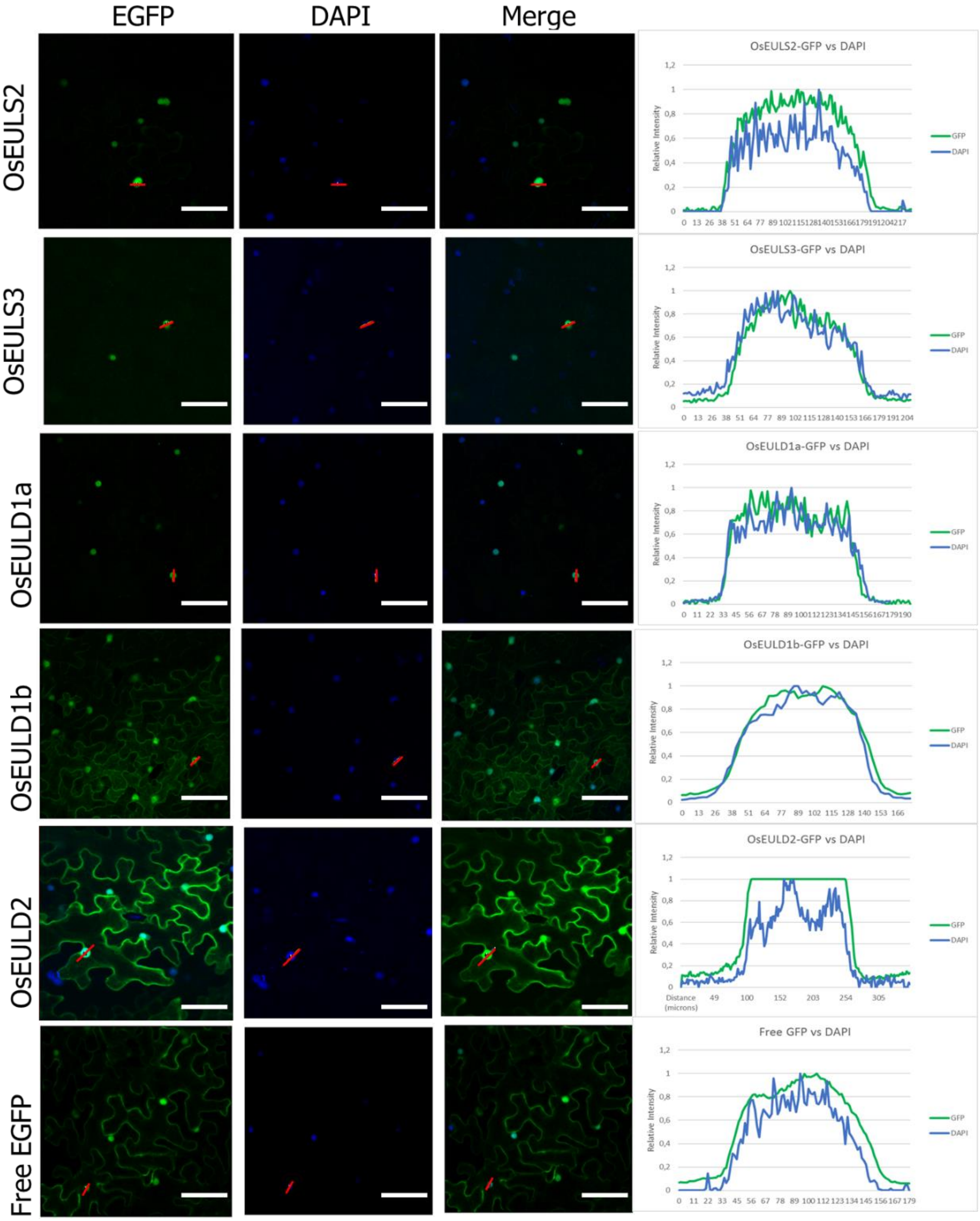

Supplementary Figure 2. Subcellular localization of OsEUL-EGFP and free EGFP in *N. benthamiana* lower epidermal cells. Nuclei were stained with DAPI. Co-localization between DAPI and EGFP signals was assessed in overlay pictures (Merge). Graphs show the plot profiles of the eGFP and DAPI fluorescent signals across the nuclei. The red line shows the part of the picture being plotted. Microscopy settings were identical for all pictures. Scale bars represent 80 µm.

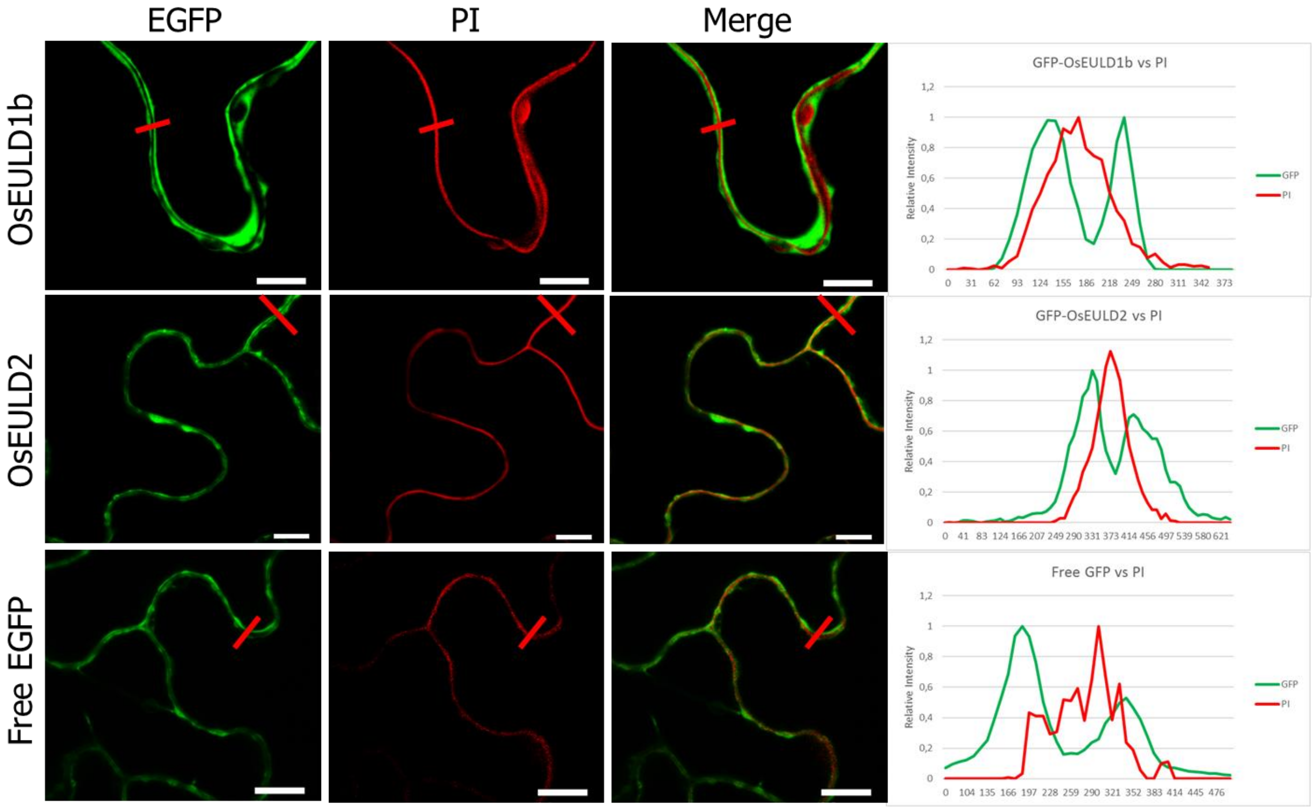

Supplementary Figure 3. Zoom in of subcellular localization of OsEULD1b, OsEULD2 and free EGFP in *N. benthamiana* lower epidermal cells. Cell walls were stained with PI. Co-localization of PI and EGFP staining was assessed in overlay pictures (Merge). Graphs show the plot profiles for eGFP and PI fluorescent signals at the border region between neighboring cells. The red line shows the part of the picture being plotted. Microscopy settings were identical for all pictures. Scale bars represent 10 µm.

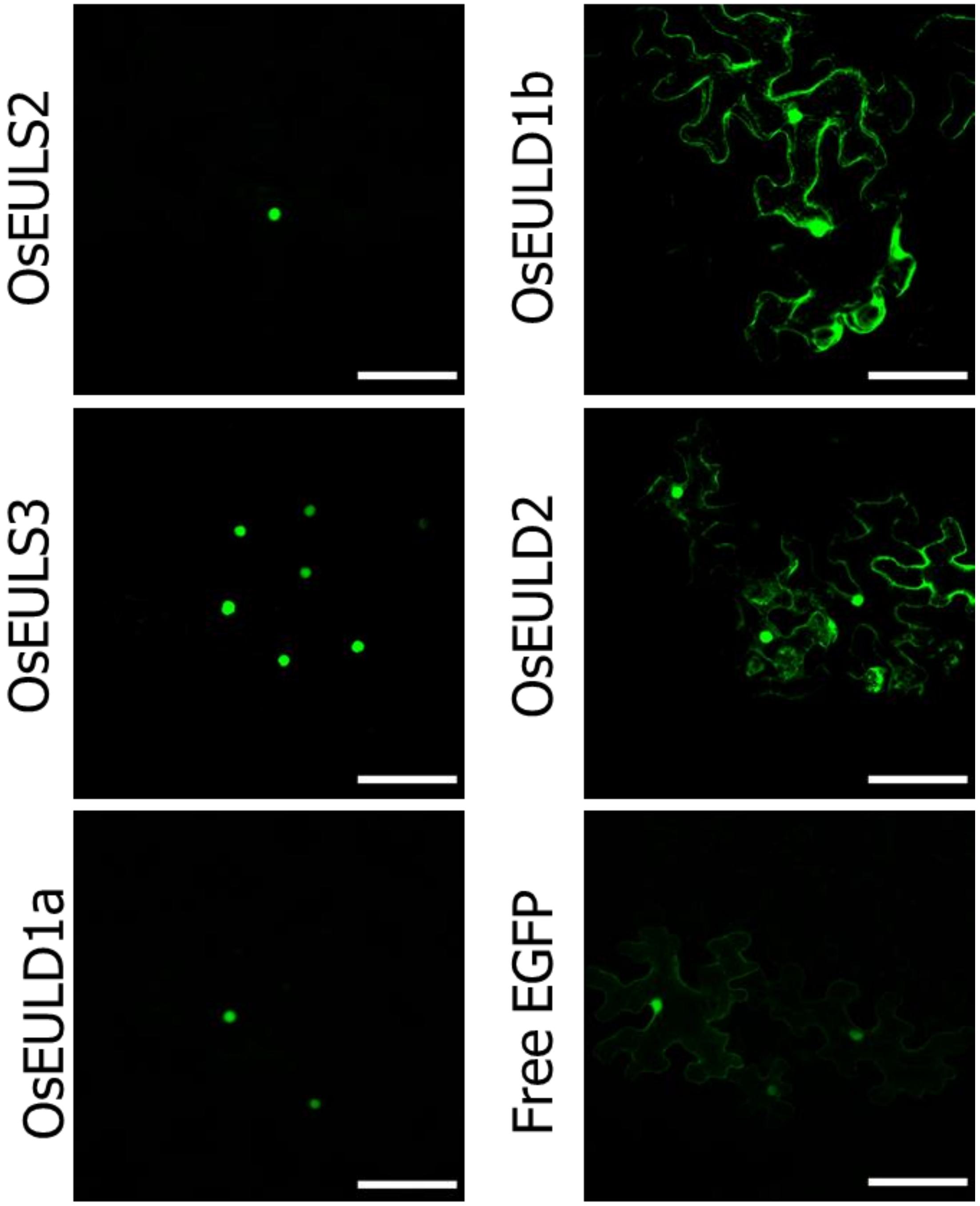

Supplementary Figure 4. Subcellular localization of OsEUL-EGFP and free EGFP in *N. benthamiana* lower epidermal cells after ABA treatment (50µM) for 1h. Microscopy settings were identical for all pictures. Scale bars represent 80 µm.

1.2 Supplementary Tables

Supplementary Table 1. Overview of gene specific primers used for RT-qPCR

Gene	LOC number	Sequence (5' to 3')	Description	Primer efficiency
Exp	LOC_Os03g27010	TGTGAGCAGCTTCTCGTTTG	forward qPCR primer to amplify a reference gene EXP	2.122
		TGTTGTTGCCTGTGAGATCG	reverse qPCR primer to amplify a reference gene EXP	
EIF5C	LOC_Os011g21990	CACGTTACGGTGACACCTTTT	forward qPCR primer to amplify a reference gene EIF5C	2.024
		GACGCTCTCCTTCTTCCTCAG	reverse qPCR primer to amplify a reference gene EIF5C	
Exp Narsai	LOC_Os07g02340	AGGAACATGGAGAAGAACAAGG	forward qPCR primer to amplify a reference gene EXPNarsai	1.981
		CAGAGGTGGTGCAGATGAAA	reverse qPCR primer to amplify a reference gene EXPNarsai	
OsEULS2	LOC_Os07g48500	CATCAAGCACTCCCTCGGCCAGTC	Forward primer for OsEULS2	1.934
		CTGAACCCGTTCCCCACGTCGCGG	Reverse primer for OsEULS2	
OsEULS3	LOC_Os01g01450	CGCTCGACTGGCCCTAGCTAAGTG	Forward primer for OsEULS3(in the 3' UTR)	1.962
		GACGTGACATGTTCATATTCGTACCG	Reverse primer for OsEULS3(in the 3' UTR)	
OsEULD1a	LOC_Os07g48490	AACAGTGGGTGATGTAAGTGCAGG	Forward primer for OsEULD1A(in the 3' UTR)	2.002
		GGGTCGAGACAAATGAGCCATTC	Reverse primer for OsEULD1A (in the 3' UTR)	
OsEULD1b	LOC_Os03g21040	CCGTGATCTGTGGAGTTGG	Forward primer for OsEULD1B (in the 3' UTR)	2.023
		GCAGGACTCGAGAAAACGAC	Reverse primer for OsEULD1B (in the 3' UTR)	
OsEULD2	LOC_Os07g48460	TCGAGAGACCGTCAACAAAA	Forward primer for OsEULD2 (in the 3' UTR)	2.023
		GGACACGCAACAGTAACACG	Reverse primer for OsEULD2 (in the 3' UTR)	
Orysata	LOC_Os01g24710	CGAAATAATGTTCCATGGTGTT	Forward primer for positive control Oryzata (in the 3' UTR)	1.999
		TGTACTACGGATCGGTGCAA	Reverse primer for positive control Oryzata (in the 3' UTR)	
WRKY45	LOC_OS05G25770	GATGGAGGACATGGAGAAGG	Forward primer for positive SA control WRKY45 (in the 3' UTR)	1.932
		GAGAAGGTGTGGAGAATCTGG	Reverse primer for positive SA control WRKY45 (in the 3' UTR)	

Supplementary Table 2. Overview of primers used in molecular cloning of the GUS reporter lines

Gene	LOC number	Sequence (5' to 3')	Description
OsEULS2	LOC_Os07g48500	CGGGGTACCATGCCCACAAA CGAGCCATAG	forward primer+KpnI site to amplify pOSEULS2
		GCCGCTCGAGGCTTCTTGATTT ATGACTCGCTAG	reverse primer+XhoI site to amplify pOSEULS2
OsEULS3	LOC_Os01g01450	CGGGGATCCGGCCGCTGATGTA TTGACTTGT	forward primer+BamHI site to amplify pOSEULS3
		GCCCTCGAGGATGGATGGATTT GGGGGAATG	reverse primer+XhoI site to amplify pOSEULS3
OsEULD1a	LOC_Os07g48490	CGGGGTACCTCCACAGGACAA CCTACACATAG	forward primer+KpnI site to amplify pOSEULD1a
		GCCGCTCGAGCCCGAACCCAA ACATCTTC	reverse primer+XhoI site to amplify pOSEULD1a
OsEULD1b	LOC_Os03g21040	CGGGGTACCGCCTTGCCTTTGA CGTGTAG	forward primer+KpnI site to amplify pOSEULD1b
		GCCGCTCGAGGTGTTGTGTGAG TGTGATGGATC	reverse primer+XhoI site to amplify pOSEULD1b
OsEULD2	LOC_Os07g48460	CGGGGTACCGACATAGCTGCTG CTCGATTG	forward primer+KpnI site to amplify pOSEULD2
		GCCGCTCGAGTCTGGTTGTCTC GCTGGTATTC	reverse primer+XhoI site to amplify pOSEULD2

Supplementary Table 3. Overview of primers used in molecular cloning of the GFP vectors

Gene	Loc number	Sequence (5' to 3')	Description
OsEULS2	LOC_Os03g21040	AAAAAGCAGGCTTCACCATGGACTTTTACGGGCGGCGCGA	forward primer for OsEULS3 CDS with non-complete attb1 site
		AGAAAGCTGGGTGTCAGTAGTAGGGCTGGATCTTCCAGCG	reverse primer for OsEULS2 CDS with non-complete attb2 site (+ stop codon for C-tag)
		AGAAAGCTGGGTGGTAGTAGGGCTGGATCTTCCAGCG	reverse primer for OsEULS2 CDS with non-complete attb2 site (- stop codon for N-tag)
OsEULS3	LOC_Os07g48460	AAAAAGCAGGCTTCTCTTCTCCCTTTTGCTATCTCTA	forward primer for OsEULS3 CDS with non-complete attb1 site
		AGAAAGCTGGGTGCCAGGGAACAATCTTCCAGC	reverse primer for OsEULS3 CDS with non-complete attb2 site (+ stop codon for C-tag)
		AGAAAGCTGGGTGCCAGGGAACAATCTTCCAGC	reverse primer for OsEULS3 CDS with non-complete attb2 site (- stop codon for N-tag)
OsEULD1a	LOC_Os07g48490	AAAAAGCAGGCTTCACCATGTTTGGGTTCGGGCACCACCA	forward primer for OsEULD1a CDS with non-complete attb1 site (+ stop codon for C-tag)
		AGAAAGCTGGGTGCTACCAGGGGAGGATCTTCCAGCGCTG	reverse primer for OsEULD1a CDS with non-complete attb2 site (+ stop codon for C-tag)
		A GAA AGC TGG GTG CCA GGG GAG GAT CTT CCA GCG CTG	reverse primer for OsEULD1a CDS with non-complete attb2 site (- stop codon for N-tag)
OsEULD1b	LOC_Os03g21040	AAAAAGCAGGCTTCACCATGTTCGGCTTCGGGCACCACGGCC	Forward primer for OsEULD1b CDS with incomplete attB1 site

		AGAAAGCTGGGTGCCAGGG GACGATCTTCCAGCGCTG	Reverse primer for OsEULD1b CDS with incomplete attB2 site (+ stop codon for C-tag)
		A GAA AGC TGG GTG TTA CCA GGG GAC GAT CTT CCA GCG CTG	Reverse primer for OsEULD1b CDS with incomplete attB2 site (-stop codon for N-tag)
OsEULD2	LOC_Os07g48460	AAAAAGCAGGCTTCACCATG TTCAGCCACCATGGGCACGG C	Forward primer for OsEULD2 CDS with incomplete attB1 site (+ stop codon for C-tag)
		AGAAAGCTGGGTGCCAGGG GAGGATCTTCCAGCGCTG	Reverse primer for OsEULD2 CDS with incomplete attB2 site (+ stop codon for C-tag)
		A GAA AGC TGG GTG TTA CCA GGG GAG GAT CTT CCA GCG CTG	Reverse primer for OsEULD2 CDS with incomplete attB2 site (-stop codon for N-tag)
AttB sites		GGGGACAAGTTTGTACAAA AAAGCAGGCT	rest of AttB1-site
		GGGGACCACTTTGTACAAGA AAGCTGGGT	rest of AttB2-site
